# Poor Dietary Quality Is Associated with Increased Inflammation in Swedish Patients with Rheumatoid Arthritis

**DOI:** 10.3390/nu10101535

**Published:** 2018-10-18

**Authors:** Linnea Bärebring, Anna Winkvist, Inger Gjertsson, Helen M. Lindqvist

**Affiliations:** 1 Department of Internal Medicine and Clinical Nutrition, Sahlgrenska Academy, University of Gothenburg, Box 459, 40530 Gothenburg, Sweden; linnea.barebring@gu.se (L.B.); anna.winkvist@nutrition.gu.se (A.W.); 2 Department of Rheumatology and Inflammation Research, Sahlgrenska Academy, University of Gothenburg, Box 480, 40530 Gothenburg, Sweden; inger.gjertsson@rheuma.gu.se

**Keywords:** diet, C-reactive protein, blood sedimentation, inflammation, rheumatoid arthritis

## Abstract

The aim was to study whether dietary quality was associated with disease activity and inflammation among patients with rheumatoid arthritis (RA). This cross-sectional analysis included 66 Swedish participants, who each completed a food frequency questionnaire (FFQ) at screening. Food intake was scored by a dietary quality index created by the Swedish National Food Agency. Disease activity was measured as Disease Activity Score 28 (DAS28), based on erythrocyte sedimentation rate (ESR), a patient administered visual analogue scale of perceived global health and the number of tender and swollen joints out of 28 examined. Inflammation was measured as ESR and C-reactive protein (hs-CRP). Associations between dietary quality, disease activity and inflammation were evaluated using multivariable linear regression analysis. High dietary quality (high intake of fish, shellfish, whole grain, fruit and vegetables and low intake of sausages and sweets) was not related to DAS28 (B = −0.02, *p* = 0.787). However, dietary quality was significantly negatively associated with hs-CRP (B = −0.6, *p* = 0.044) and ESR (B = −2.4, *p* = 0.002) after adjusting for body mass index, age, education, smoking and gender. Both hs-CRP and ESR decreased with increasing dietary quality. In conclusion, among patients with RA, high dietary quality was associated with reduced inflammation but not with disease activity.

## 1. Introduction

Rheumatoid Arthritis (RA) is a chronic autoimmune disease that affects 0.5–1% of the Western population. In Sweden, the incidence of RA is about 41 per 100,000 [1]. The disease is characterized by painful joint inflammation, which causes irreversible joint destruction, disabilities and reduced quality of life. Pharmacological treatment of RA has become much more effective, but approximately only half of the patients with newly diagnosed RA in 2016 in Sweden reached remission, i.e., no active disease [2]. This indicates that other treatment modalities, alongside the pharmacological treatment, should be explored in order to decrease disease activity and inflammation in patients with RA. 

A number of individual factors such as age, gender, body mass index (BMI), smoking and diet can influence and modify the inflammatory response [3]. Diet has the potential to decrease inflammation and disease activity in RA, through several pathways: e.g., dietary fatty acids influence the production of cytokines and eicosanoids, and a reduced intake of arachidonic acid and an increased intake of long chain omega three fatty acids, can ameliorate symptoms of RA [4]. Additionally, antioxidant intake might reduce the level of inflammation markers by reducing oxidative stress [5,6]. Further, gut microbiota might mediate inflammatory responses and may itself be improved by pre- and probiotics [7,8] which indicates a role for dietary fiber. 

Gender, higher BMI and age are associated with higher disease activity and inflammation in RA [9]. Higher BMI and smoking are also risk factors in developing RA [10,11]. Further, being overweight at diagnosis is associated with a decreased chance of adequate disease control [12]. In addition, research indicates that poor dietary quality may be an independent risk factor in developing RA. For example, intake of sugar sweetened soda [13] is associated with an increased risk while long chain n-3 fatty acids [14], vegetables and dairy [15], and the Mediterranean dietary pattern [16] are associated with decreased risk. However, associations between diet and the risk of developing RA have not been confirmed in all studies [17,18]. 

In established RA, diet may be of importance for disease progression. Regrettably, patients with RA have unsatisfactory dietary quality, and poorer diet than healthy controls [19,20]. Poor dietary quality is associated with longer lasting morning stiffness [19] and more functional disabilities [20]. Other studies have shown that higher disease activity is associated with lower intake of fish oil, monounsaturated fatty acids [21] and n-3 polyunsaturated fatty acids [22]. Few intervention studies have investigated dietary treatment for decreasing disease activity and inflammation in RA, but some positive results have been noted for the Mediterranean diet [23,24], gluten free vegan diet [25] and fasting followed by vegan and vegetarian diet [26,27]. Overall, there are indications that dietary intake could modify disease activity and inflammation in RA. Possibly, a higher intake of dietary fiber (plant-based foods such as fruit and vegetables and whole grain) and a high-quality fat intake (lower intake of saturated fats and a higher intake of unsaturated and long-chain polyunsaturated fatty acids) could contribute to decreased inflammation and disease activity. However, despite indications from patients with RA that dietary intake impacts the disease [28], there is a great lack of research in this area.

The aim of this cross-sectional study was to determine whether dietary quality was associated with disease activity and inflammation among patients with RA in Sweden.

## 2. Materials and Methods 

Participants were recruited to the randomized cross-over trial ADIRA (Anti-inflammatory Diet in Rheumatoid Arthritis) [29] during 2017 and the data presented here are based on the initial screening visit. The study was registered at Clinical Trials prior to recruitment (NCT02941055). Patients in the region of Västra Götaland, Sweden, were identified through the Swedish Rheumatology Quality Registry. In total, 1091 patients (244 men, 847 women) were considered eligible and 774 patients resided within reasonable distance from the Sahlgrenska University Hospital, Gothenburg, Sweden, and were therefore invited to participate. Inclusion criteria for the intervention study were age >18 years, disease duration ≥2 years, stable pharmacological treatment during the last 3 months and a willingness to consume an omnivore diet. Exclusion criteria were other serious illness, longer travel time, planned surgery, pregnancy and a food allergy or avoidance of foods included in the intervention study. A total of 113 individuals showed interest in participating in the study. Those (*N* = 47) who did not fulfil the inclusion criteria after initial contact were excluded. A total of 66 patients were screened during a visit to the Sahlgrenska University Hospital and were included in the present cross-sectional analysis. This study was conducted according to the Declaration of Helsinki and all procedures were approved by the Regional Ethics Committee in Gothenburg (No. 976-16, of November 2016). Written and informed consent was provided by all participants.

Food intake at baseline was reported by a 53-item food frequency questionnaire (FFQ), reflecting dietary intake during the last 12 months. Overall diet quality was calculated by a dietary quality index created by the Swedish National Food Agency [30], based on the dietary recommendations in the Nordic Nutrition Recommendations [12]. Briefly, dietary quality is scored by consumption frequency of the following foods; fruit and vegetables, whole grain bread, fish and shellfish, fat spreads, cheese (24–40% fat), sausage and discretionary foods (sweets, cakes, soft drinks and fried potatoes). Frequent consumption of fruit, vegetables, whole grain bread and fish contribute positively to the index score, while intake of cheese, sausage and discretionary foods contribute negatively. Habitual use of low-fat spread (≤40%) contributes positively while high fat spread (≥60%) contributes negatively. The index is an indicator of dietary quality (intake of fat, sugar and fiber) and compliance with dietary recommendations (intake of fruit, vegetables, fish and whole grain bread). The total score ranges from 0 to 12, and dietary quality considered low at 0–4 points, fair at 5–8 points and high at 9–12 points [30].

Disease activity was defined by Disease Activity Score 28 (DAS28), which is a composite score based on the examination of 28 joints in combination with the erythrocyte sedimentation rate (ESR) and the patients’ perceived health on a visual analogue scale. ESR and high sensitivity C-reactive protein (hs-CRP) were measured at the routine laboratory for Clinical Chemistry at Sahlgrenska University Hospital. BMI was calculated using weight and height measured at the screening visit. Data on education and smoking were collected using patient administered questionnaires. Education level was classified into primary level, secondary level or university level. Smoking referred to current use and was coded as a binary variable (yes/no). 

The ADIRA randomized cross over trial needed 38 participants to detect a significant difference of 0.6 units of DAS28 between the diet groups (80% power), and screened 66 individuals. Based on observational dietary data among individuals at cardiometabolic risk [31], a group size of 44 would yield sufficient statistical power to detect differences in hs-CRP (6 vs. 3 ± 5 mg/L) between those with high and poor dietary quality (80% power, alpha 0.05).

Gender differences in dietary quality and categorical participant characteristics were studied using the Chi-square test. Gender differences in continuous participant characteristics were studied using the Mann Whitney-U test and Student’s *t*-test. Dietary quality was studied in relation to DAS28, hs-CRP and ESR using multivariable linear regression analysis. Dietary quality was used both as a continuous variable (0–12) and classified into poor (0–4), fair (5–8) and high (9–12) quality. DAS28 was categorized as remission (<2.6), low disease activity (2.6–3.2) and moderate-high disease activity (>3.2). Both crude and adjusted models are presented, and all multiple models are adjusted for age, BMI, education level, gender and smoking. Statistical analyses were performed using software SPSS Statistics 25.0 (IBM). Significance was accepted at *p* < 0.05. 

## 3. Results

The age ranged from 27–74 years and the study included subjects from normal weight to obesity. In total, 80% of the participants were female, 55% were educated at university level and 5% smoked (Table 1). A total of 26% of the participants were in remission (DAS28 < 2.6), 21% had low disease activity (DAS28 2.6–3.2), 49% had moderate disease activity (DAS28 3.2–5.1) and 5% had high disease activity (DAS28 > 5.1).

The median (Q1–Q3) dietary index score was 6.5 (5.0–7.0) points, and ranged from 0 to 10 points. The median dietary index scores among women and men were 6.4 and 5.5, respectively (*p* = 0.074). Most participants reported consuming fruit and vegetables 1–2 times/day and most reported consuming fish and shellfish 3–6 times/week (Table 2). Margarine (≥60% fat) or butter as spread was favored by 73% of the participants while 27% reported using low fat margarine (≤40% fat). Dietary quality was classified as fair for most participants (76%), while 15% and 9% had a poor or high-quality diet, respectively. Women reported more frequent intake of fruit and vegetables than did men, but no other significant gender differences in reported dietary intake were noted (Table 2). There was a tendency towards younger age among participants with poor dietary quality (Table 1).

Neither the dietary index score nor the dietary quality was related to DAS28 in unadjusted or adjusted regression analyses (Table 3). In the multivariable linear regression model (Table 3), higher dietary quality was significantly associated with lower hs-CRP. Higher dietary quality was also significantly associated with lower ESR (Table 3). When dividing dietary quality into three groups, poor quality diet was associated with higher ESR (B = 16.794, *p* = 0.005) and CRP (B = 5.018, *p* = 0.031) compared to a high-quality diet, in adjusted analyses. The associations between dietary quality and inflammation seemed to be driven by the indicators of fiber intake and fat quality, and less by intake of discretionary foods (Table 3).

There were no significant associations between dietary quality and VAS general health (B = 1.797, *p* = 0.236), tender (B = 0.354, *p* = 0.190) or swollen joint counts (B = 0.158, *p* = 0.186) in adjusted analyses. 

## 4. Discussion

In this study, we showed that, in patients with RA, higher dietary quality, as assessed by a dietary index based on the Swedish dietary guidelines, was associated with lower hs-CRP and ESR. However, there were no significant associations between dietary quality and disease activity, measured as DAS28. 

Disease activity in RA is often measured as DAS28, which is a composite score that consists of both objective and subjective measures. The patients included in this study had a mean DAS28 of 3.4, which is considered moderate disease activity [32]. The results from our study indicated that the measures of inflammation were more noticeably associated with dietary quality than were the measures of general health and joint status. This could possibly be explained by greater variation in measures of inflammation than joint status, and that global health is a subjective measure affected by a variety of other aspects relating to wellbeing. A previous study has related dietary quality to morning stiffness, which normally correlates with DAS28, but not to hs-CRP in adjusted analyses [19]. This previous study was conducted among 84 American women and men with RA, and used the more complex Healthy Eating Index to assess dietary quality. Still, the American study did not adjust for confounders, apart from BMI, which may explain the disparate results. 

The dietary index used in this study was constructed to capture the quality of dietary fat by assessing intake of fish and shellfish, spread margarine, cheese and sausage and quality of dietary carbohydrate by assessing intake of fruit and vegetables, whole grain bread and discretionary foods (including sweets, biscuits, soft drinks, lemonade and fried potatoes). The index was developed by the Swedish National Food Agency [30], and provided an estimate of dietary quality in relation to the evidence based dietary guidelines in the Nordic Nutrition Recommendations [12]. The index was therefore likely to have provided a relevant estimate of dietary quality. This was, to our knowledge, the first time it had been studied in relation to RA. 

The dietary quality was fair for most participants but less than 10% were classified as having a high-quality diet. Hence, our sample of patients with RA had similar dietary quality compared to the general population in Sweden, where approximately 10% had a high-quality diet and an average score of approximately six points in 2008 [30]. However, the intake of fish seemed more frequent among our patients with RA, as 73% reported eating fish or shellfish at least two times/week, compared to 38% in the general population [30]. Additionally, 69% of the general population reported eating fruit and vegetables less frequently than three times/day, compared to only 44% of patients with RA. The patients with RA also reported a lower intake of discretionary foods compared to the general population. This was in line with previous data, indicating that patients with RA report that these foods worsen their symptoms [28] and they therefore might avoid them. Additionally, the patients with RA had all volunteered to participate in a dietary intervention trial, and might therefore have been more interested in diet than the general population. The dietary data from the general population were also collected in 2008, and dietary changes over time could be another explanation for any disparities in the comparisons with patients with RA. Education level and smoking were not associated with dietary quality in our analyses, perhaps due to lack of statistical power as few patients smoked and few had the highest completed education at primary level. 

### Strengths and Limitations

This was a cross sectional analysis and could therefore not evaluate causal relations between diet and inflammation or disease activity in RA. The small number of participants was a limitation, as was our inability to adjust for total energy intake in the present analyses. It is possible that adjusting the intake data for total energy intake may have weakened the associations found. Still, by adjusting for BMI, age and gender, the majority of variation in energy intake was likely captured. Further, only patients with an omnivore diet and who did not have food allergies or intolerances were included in the study. Inclusion of all patients with RA, despite dietary habits and avoidances, might have added an extra dimension to this work. The strengths of this study were that the patients were derived from a population-based register of high quality. In addition, the patients included in this study seemed representative of patients with RA in Sweden, though they had a slightly higher education level [1]. The diet quality index used in this study was developed by the Swedish National Food Agency, thus covering patient-relevant aspects of a healthy diet and allowing comparison with national results. 

## 5. Conclusions

Higher dietary quality in patients with RA who follow an omnivorous diet was associated with lower hs-CRP and ESR. However, there was no association between dietary quality and disease activity, measured as DAS28. Well-designed dietary intervention trials are needed to confirm that a high-quality diet can reduce inflammation in patients with RA. 

## Figures and Tables

**Table 1:** Characteristics of the 66 Swedish patients with rheumatoid arthritis.

	**All** ***N* = 66**		**Poor Dietary** **Quality (*N* = 10)**		**Fair Dietary** **Quality (*N* = 50)**		**High Dietary** **Quality (*N* = 6)**		
	**Mean**	**SD**	**Mean**	**SD**	**Mean**	**SD**	**Mean**	**SD**	***p*^a^**
**Dietary quality**	6.2	1.9	3.1	1.2	6.5	1.1	9.5	0.6	<0.001
**Age (years)**	59.9	12.2	52.8	13.0	60.6	12.1	65.9	8.1	0.096
**BMI (kg/m^2^)**	27.6	5.1	26.1	5.1	28.0	5.0	26.2	5.7	0.412
**Waist hip ratio**	0.86	0.08	0.90	0.10	0.86	0.08	0.83	0.05	0.325
**DAS28 (ESR)**	3.4	1.1	3.3	0.9	3.5	1.1	2.9	1.3	0.528
**hs-CRP**	3.9	5.0	6.4	6.2	3.7	4.9	1.8	1.7	0.066
**ESR**	17.5	11.7	22.5	9.8	17.3	12.2	10.8	7.2	0.105
**General Health VAS (mm)**	36.5	22.4	31.0	17.5	37.1	22.3	40.0	32.3	0.831
**Tender joint count**	3.36	3.9	1.8	2.4	3.8	4.2	2.3	3.3	0.172
**Swollen joint count**	1.7	1.7	1.2	1.2	1.8	1.8	1.3	2.0	0.499
	**All** ***N* = 66**		**Poor Dietary** **Quality (*N* = 10)**		**Fair Dietary** **Quality (*N* = 50)**		**High Dietary** **Quality (*N* = 6)**		***p*^b^**
**Education level**	**N**	**%**	**N**	**%**	**N**	**%**	**N**	**%**	0.589
Primary level	10	15	1	10	9	90	0	0	
Secondary level	20	30	4	20	13	65	3	15	
University level	36	55	5	14	28	78	3	8	
**Smoking**									0.605
No	63	95	9	14	48	76	6	10	
Yes	3	5	1	33	2	67	0	0	
**Gender**									0.342
Female	53	80	7	13	40	75	6	11	
Male	13	20	3	23	10	77	0	0	

^a^ Derived from Kruskal Wallis test, ^b^ Derived from Chi square test; BMI, body mass index; DAS28, disease activity score 28-joints; ESR, Erythrocyte sedimentation rate; VAS, visual analogue scale.

**Table 2:** Dietary intake of the 66 Swedish patients with rheumatoid arthritis.

	Percent, All (*N* = 66)	Percent, Women (*N* = 53)	Percent, Men (*N* = 13)	*p*
**Dietary quality**				0.342
Poor (0–4 points)	15	13	23	
Fair (5–8 points)	76	76	77	
High (9–12 points)	9	11	0	
**Fruit and vegetables ^a^**				<0.001
<3 times per day	44	32	92	
3–4 times per day	49	59	8	
≥5 times per day	8	9	0	
**Whole grain bread ^b^**				0.663
<1/day	32	32	31	
1–2 /day	53	55	46	
≥3/day	15	13	23	
**Fish and shellfish**				0.168
<1 times/week	11	8	23	
1–2 times/week	17	15	23	
≥2 times/week	73	77	54	
**Discretionary foods ^c^**				0.634
>7 times/week	14	13	15	
3–6 times/week	26	28	15	
<3 times/week	61	59	69	
**Spread margarine ^d^**				0.705
≥60%	73	72	77	
≤40%	27	28	23	
**Cheese 24–40% fat**				0.952
≥4 times/week	61	60	62	
1–3 times/week	21	21	23	
≤1 times/week	18	19	15	
**Sausage ^e^**				0.890
>1 times/week	17	17	15	
≤1 times/week	83	83	85	

^a^ Not including fruit juice, ^b^ Hard bread and whole grain bread, ^c^ Sweets, cakes, soft drinks, lemonade, fried potatoes, ^d^ Includes margarine and butter, ^e^ Sausage and sausage dishes.

**Table 3:** Associations between dietary quality and Disease Activity Score 28 (DAS28), high sensitivity C-reactive protein (hs-CRP) and erythrocyte sedimentation rate (ESR) among 66 Swedish patients with rheumatoid arthritis.

	DAS28			hs-CRP			ESR		
	Beta	Std. Err	*p*	Beta	Std. Err	*p*	Beta	Std. Err	*p*
**Unadjusted model**									
Dietary index score (0–12)	−0.028	0.073	0.707	−0.560	0.321	0.086	−1.700	0.743	0.025
**Adjusted model ^a^**									
Dietary index score (0–12)	−0.020	0.072	0.787	−0.607	0.295	0.044	−2.420	0.743	0.002
**Dietary quality indicators ^a^**									
Fiber intake score (0–4) ^b^	−0.198	0.146	0.182	−1.514	0.622	0.018	−2.301	1.107	0.042
Fat quality score (0–6) ^c^	0.093	0.100	0.359	−0.847	0.429	0.053	−3.545	1.624	0.033
Sugar intake score (0–2) ^d^	−0.109	0.172	0.526	0.545	0.752	0.472	−3.286	1.909	0.090

^a^ Adjusted for age, body mass index, education level, smoking and gender. ^b^ Sum of score from fruit, vegetables and whole grain bread. ^c^ Sum of score of spread margarine, cheese, sausage, fish and shellfish. ^d^ Sweets, cakes, soft drinks, lemonade, fried potatoes.
